# Idiopathic Superior Mesenteric Vein Thrombosis Resulting in Small Bowel Ischemia in a Pregnant Woman

**DOI:** 10.1155/2011/687250

**Published:** 2011-09-14

**Authors:** Hao Lin, Chih-Che Lin, Wan-Ting Huang

**Affiliations:** ^1^Department of Obstetrics and Gynecology, Kaohsiung Chang Gung Memorial Hospital and Chang Gung University College of Medicine, Kaohsiung City 833, Taiwan; ^2^Department of Surgery, Kaohsiung Chang Gung Memorial Hospital and Chang Gung University College of Medicine, Kaohsiung City 833, Taiwan; ^3^Department of Pathology, Kaohsiung Chang Gung Memorial Hospital and Chang Gung University College of Medicine, Kaohsiung City 833, Taiwan

## Abstract

*Background*. Small bowel ischemia due to superior mesenteric vein thrombosis (MVT) is rare during pregnancy. However, additional precipitating factors should usually be identified. *Case*. A 31-year-old woman, pregnant at 34 weeks, was sent to the emergency department because of acute peritonitis. An emergency exploration revealed a segmental gangrene of the small intestine without any mechanical obstruction. Together with the termination of pregnancy, resection of the damaged small bowel was performed, and an end-to-end enterostomy was followed. Based on the operative and pathological findings, small bowel ischemia might be attributed to superior mesenteric vein thrombosis. *Conclusion*. Hypercoagulation state normally found in pregnant women is believed to lead to this catastrophic condition without other precipitating factors.

## 1. Introduction

Small bowel ischemia is a relatively uncommon disorder; the primary causes are diverse and can be grouped into five major categories: (1) strangulation, (2) low-flow states (arrhythmia, sepsis, shock), (3) embolus or thrombosis of superior mesenteric artery, (4) superior mesenteric vein thrombosis (MVT), and (5) miscellaneous. Due to vague clinical presentation and the lack of specific diagnostic tests, early diagnosis for intestinal ischemia is difficult, resulting in significant morbidity and mortality. We herein report a case of a 31-year-old woman who was presented with the signs and symptoms of peritonitis at 34-week gestation. Surgical exploration revealed gangrene in the small intestine without mechanical obstruction. Hypercoagulable state is normally found in pregnant women, which was believed to result in superior MVT and then intestinal ischemia. To the best of our knowledge, this is the second report to describe such a rare disease developing during pregnancy, without evidence of other precipitating factors.

## 2. Case Report

**Figure 1:**
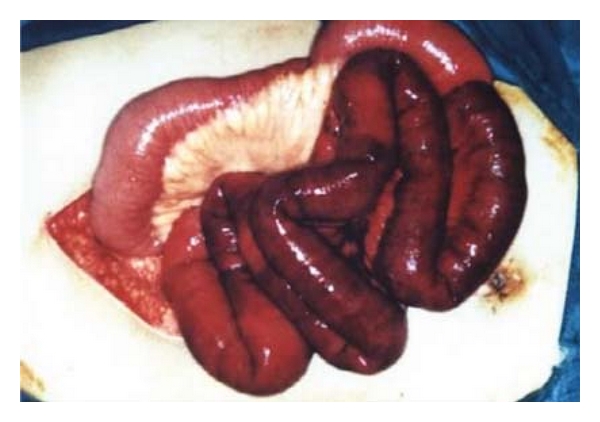
Segmental gangrene of the small intestine without any obstruction and perforation.

**Figure 2:**
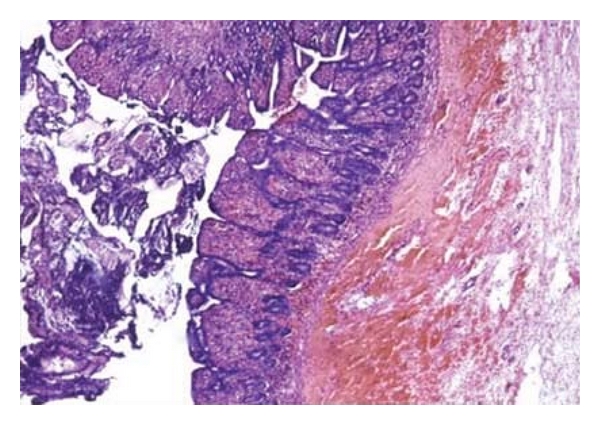
Presence of well-formed surface exudates of neutrophils and fibrin (left) with mucosal and submucosal hemorrhage (right) to reveal the ischemic change of small bowel. (H&E stain, 100x).

At 34 weeks of gestation, a 31-year-old gravida 3 para 1 woman was referred to our emergency department from a local clinic owing to progressive epigastric pain associated with nausea and vomiting for 3 days. She had no significant medical or surgical history and no history suggestive of thromboembolism. She had never used any oral contraceptives or other hormonal preparations. Upon arrival, her vital signs were stable without fever (BP 120/78 mmHg, pulse rate 88/min, respiratory rate 19/min, and body temperature 37.1°C). The hematologic examination revealed marked leukocytosis (WBC 24,200/CMM) and relative intravascular depletion (hematocrit 44.5%). Coagulation profile and biological tests were within normal limits. Obstetric ultrasound showed a normal male fetus compatible with his gestational age. In addition, a mild fatty change in the liver and a moderate amount of ascites were also noted. The fetal monitor showed that the uterus contracts every 10 minutes. She was initially kept for conservative treatment (fasting, nasogastric suction) and absolute bed rest under the suspicion of acute gastroenteritis and preterm labor. However, signs of acute peritonitis gradually developed within 6 hours after admission. Thus, an emergency exploration was performed to find a segmental gangrene of the small intestine with 1,500 mL of serosanguineous peritoneal fluid, but no frank obstruction and perforation was identified (Figure 1). The premature baby, at 1,850 grams, was then delivered via cesarean section, with an Apgar score of 4 at one minute and 6 at five minutes. The gangrenous small intestine was resected, and a primary anastomosis was performed. Her postoperative course was uncomplicated, and she was discharged one week after surgery. The outcome of the baby was also excellent without any sequela after intensive care. The pathologic examination revealed mucosal denudation, submucosal edema and hemorrhage, and inflammatory infiltration in the muscularis propria. The intramural and mesenteric vessels in the specimen were patent with blood stasis (Figure 2). Screens for inherited thrombotic disorders (protein S, protein C, antithrombin III deficiency, and factor V Leiden mutation) for the patient were done after surgery to demonstrate negative results. Four years later, the patient was pregnant again and delivered at term without any thromboembolic event throughout the course. Till now she has been recurrence-free for 7 years without anticoagulant therapy. 

## 3. Discussion

Small bowel ischemia occurs very infrequently during pregnancy or puerperium. After searching the MEDLINE, only 16 cases of small bowel ischemia are reported to demonstrate the association of its occurrence with pregnancy to date. Among them, 5 cases (31.2%) were caused by the volvulus of the bowel [1–5], one (6.3%) by diverticulitis [6], one (6.3%) was attributed to cocaine abuse [7], and the remaining 9 cases (56.2%) were induced by superior MVT [8–16]. Generally an uncommon type of small bowel ischemia, superior MVT, precipitates more than half of the cases in pregnant women. Precipitating factors associated with MVT may include portal hypertension, postoperative state, trauma, neoplasm, inflammatory bowel diseases, use of estrogen-containing compounds, polycythemia vera, hemoglobinopathies, and hypercoagulable states resulting from protein S, protein C, and antithrombin III deficiencies [17]. Through the literature review, additional precipitating factors could be identified in 8 of the 9 pregnant women with small bowel ischemia due to superior MVT: 2 had mistakenly used oral pills during pregnancy [8, 9], 2 had known history of hypercoagulopathies [10, 11], 1 developed MVT soon after elective cesarean section [12], 1 had CMV infection complicated further by toxic megacolon [13], 1 had history of chronic idiopathic MVT [14], and 1 had hemoglobinopathy [15]. Only one case in the literature and our case possess no precipitating factor to develop MVT [16]. 

Early diagnosis of superior MVT is very difficult due to the nonspecific signs, symptoms, and laboratory results. Almost all patients present with abdominal pain, nausea and vomiting, and leukocytosis with elevated hematocrit, but these are not helpful for diagnosis [17]. Today, the diagnosis of superior MVT is established by a high index of clinical suspicion and noninvasive imaging of the abdomen by ultrasonography, computed tomography, or magnetic resonance imaging (MRI) [18]. The correct diagnosis can be confirmed at laparotomy. Most patients undergo segmental resection of the bowel because of late diagnosis and immediate anticoagulant therapy. The microscopic features of superior MVT include hyperemia, hemorrhage, and edema of the intestinal wall. Mucosal destruction alone occurs in early and less severe lesions, and transmural infarction occurs in the most severe cases. Fresh and organized thrombi extrude from the veins, while arteries are constricted but patent [19]. Although our patient had a similar clinical presentation and laboratory findings, superior MVT was not included in our initial differential diagnosis. Therefore, we did not focus on the mesenteric venous flow or thrombus while performing ultrasonography. Diagnosis was made at laparotomy with the appearance of typical segmental gangrene of the small intestine in association with serosanguineous peritoneal fluid [18]. Microscopic findings of the specimen were typical, except that no thrombi were identified. Whether or not the “presence of thrombi” is essential for the diagnosis of superior MVT remains controversial and requires further investigation.

Once the diagnosis of superior MVT is made, the immediate use of anticoagulation is indicated due to the high recurrence rate. In patients with inherited hypercoagulable disorders (i.e., protein S, protein C, antithrombin III deficiencies, and factor V Leiden mutation), life-long anticoagulation is warranted. For patients with reversible predisposing causes, at least 6 months of anticoagulation is recommended [18]. Though our patient has been recurrence-free for 7 years after surgery without anticoagulation, the need of prophylactic anticoagulant in this idiopathic etiology still needs further investigation. 

In conclusion, it is suggested that in a pregnant woman with a clinical presentation of acute abdomen, even with the absence of the other known risk factors, superior MVT should be included in the differential diagnosis because the pregnancy itself should be considered as an important background etiological factor.
